# Pedagogical Practices That Enhance Medical Students’ Capacity for Creative Thought: A Qualitative Study

**DOI:** 10.1192/bjo.2025.10149

**Published:** 2025-06-20

**Authors:** Muhammad Talha Zaigham, Zuraiz Idrees

**Affiliations:** 1 Ysbyty Cwm Cynon, Mountain Ash, United Kingdom; 2 Portsmouth Hospitals University NHS Trust, Portsmouth, United Kingdom

## Abstract

**Aims:** In the medical field, there is a growing emphasis on fostering creativity and innovation in medical students to prepare them for the unpredictable nature of patient care. This study aimed to explore the perspectives of both lecturers and medical students on the current teaching practices and their influence on the development of creative thinking skills.

**Methods:** The study was conducted as qualitative research at the Malaysian Faculty of Medicine and Health Sciences and included a purposeful sample of eight medical students and seven lecturers. Data were gathered through individual semi-structured interviews held via the Google Meet platform and analysed using a thematic analysis approach.

**Results:** The findings indicate that learner-centred approaches, such as problem-solving exercises and group discussions, seminars, debates, and dramas have a positive impact on enhancing their creative thinking abilities. The use of technology-assisted teaching methods, including e-learning and simulation labs, was also perceived as inspiring, however, limitations in technical infrastructure were noted. Challenging activities like assignments, games, competitions, and online tests encourage creative learning. Hands-on activities, such as bedside teaching and clinical skill learning, are also valuable in learning clinical skills in unique ways, but their effectiveness could be reduced by environmental and personal factors. Furthermore, practicing para-curriculum activities in a supportive and relaxed learning environment was identified as fostering a culture of original thought.

**Conclusion:** This study suggests that a comprehensive approach to medical education that integrates creative pedagogy can be instrumental in fostering creativity in medical students. Providing opportunities for creative thinking through workshops and addressing technical infrastructure limitations in technology-assisted teaching methods could be considered in enhancing the creative curricula in the South East region. The findings underline the importance of a learner-centred approach and a supportive learning atmosphere in promoting creative learning.

